# Rifampicin‐Associated Sweet Syndrome: An Uncommon Adverse Event of Anti‐Tuberculosis Therapy

**DOI:** 10.1002/rcr2.70414

**Published:** 2025-11-20

**Authors:** Sze Kye Teoh, Yen Shen Wong, Nai Lim Lai, Nor Azila Md Akil, Syarifah Nabilah Syed Junid Aljunid, Yu Wei Cheah, Saiful Safuan Md Sani

**Affiliations:** ^1^ Department of Medicine Hospital Ampang Ampang Malaysia; ^2^ Faculty of Medicine University Technology MARA Sungai Buloh Malaysia; ^3^ Department of Dermatology Hospital Ampang Ampang Malaysia; ^4^ Department of Pathology Hospital Sultan Idris Shah Serdang Malaysia

**Keywords:** adverse drug reaction, antituberculous therapy, neutrophilic dermatosis, rifampicin, Sweet syndrome

## Abstract

Sweet syndrome, or acute febrile neutrophilic dermatosis, is an uncommon inflammatory condition that may arise secondary to infection, malignancy, autoimmune disease, or drugs. Its association with tuberculosis is rare, and rifampicin‐induced Sweet syndrome has been infrequently reported. We present a 58‐year‐old man with disseminated tuberculosis who developed painful erythematous plaques shortly after commencing antituberculous therapy (ATT). The eruption improved with corticosteroids but recurred specifically on rechallenge with rifampicin, confirming a drug‐induced aetiology. Rifampicin was excluded, and the patient completed ATT successfully with the remaining first‐line drugs. Rifampicin‐induced Sweet syndrome is a rare but important differential in patients who develop erythematous plaques on ATT. Accurate diagnosis allows continuation of essential tuberculosis treatment while avoiding unnecessary discontinuation of other first‐line drugs.

## Graphical abstract

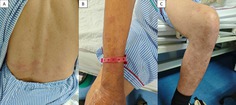

## 1 Introduction

Sweet syndrome, or acute febrile neutrophilic dermatosis, is characterised by painful erythematous plaques, fever, neutrophilia, and dense dermal neutrophilic infiltrates. It is typically idiopathic but may occur secondary to infection, malignancy, autoimmune disease, or drug exposure [1, 2, 3]. Tuberculosis‐associated Sweet syndrome is rare, with rifampicin‐induced cases reported only sporadically [4, 5].

## 2 Case Report

A 58‐year‐old man with hypertension presented with a three‐month history of a penile plaque. Clinical evaluation and imaging revealed generalised lymphadenopathy. Biopsies of the penile lesion and inguinal lymph nodes showed necrotising granulomatous inflammation, and interferon gamma release assay was positive, confirming disseminated tuberculosis.

**FIGURE 1:**
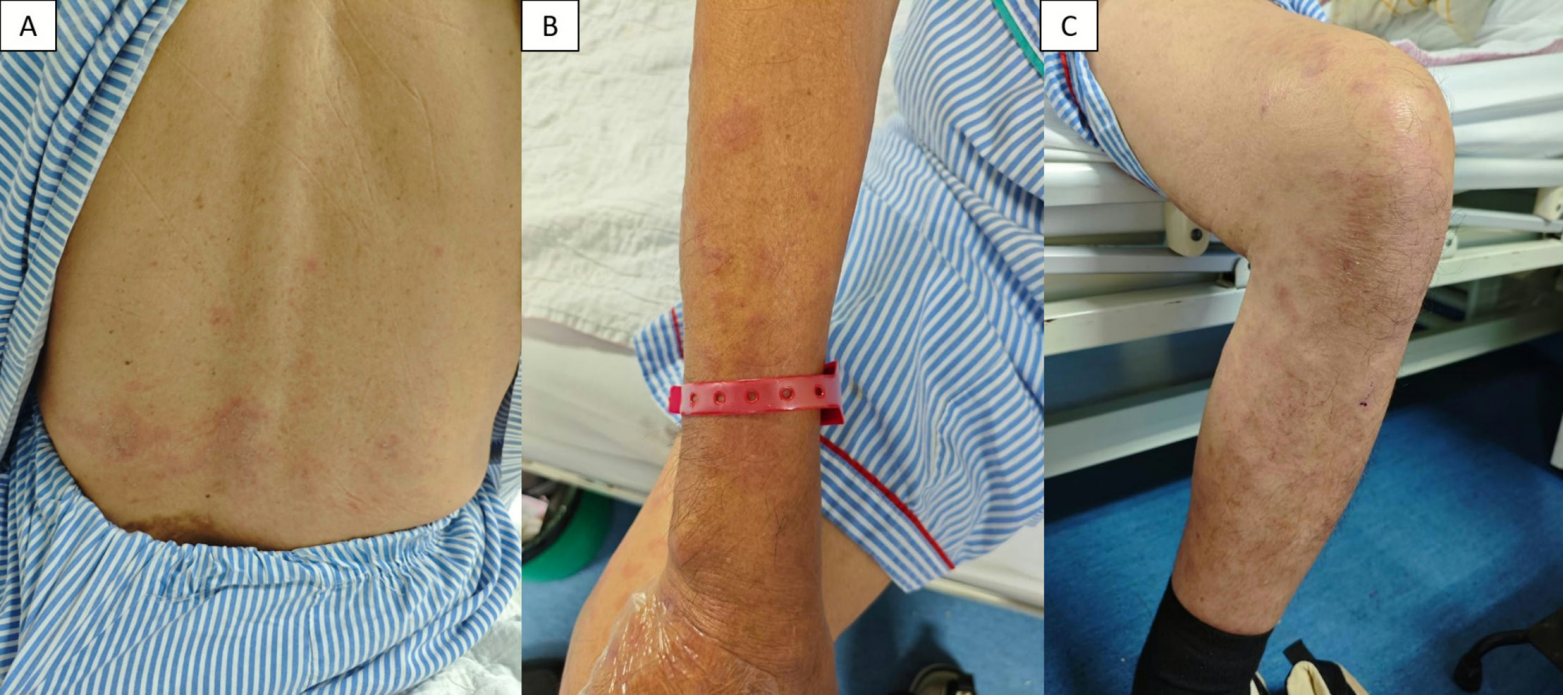
Cutaneous findings after 2 weeks of anti‐tuberculous therapy. Multiple painful erythematous plaques over the back (A), upper limbs (B) and lower limbs (C).

He was started on standard ATT (isoniazid, rifampicin, pyrazinamide, ethambutol). Within 1 week, he developed multiple painful erythematous plaques over the upper and lower limbs, associated with systemic malaise Figure 1. ATT was withheld, with partial resolution of the lesions. Rechallenge with the full regimen caused worsening of cutaneous lesions.

**FIGURE 2:**
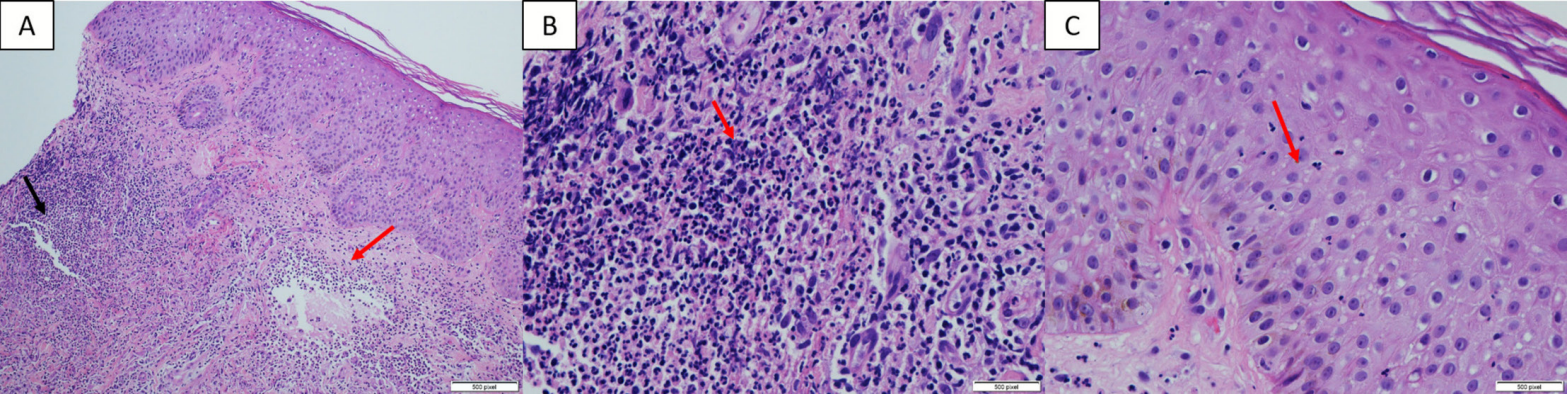
Histopathological findings of skin biopsy. (A) A dense neutrophilic dermal infiltrate in the papillary dermis is associated with papillary oedema. (B) Dermal papillary microabscesses are seen within the papillary dermis. No granulomatous or vasculitic features were present. (C) Mild epidermal spongiosis with neutrophilic exocytosis was observed.

Skin biopsy demonstrated spongiotic dermatitis with dense dermal neutrophilic infiltrates, consistent with Sweet syndrome Figure 2. Corticosteroids provided marked improvement and stepwise drug re‐challenge identified rifampicin as the culprit, as lesions reappeared only on re‐exposure. Rifampicin was excluded, and the patient successfully completed ATT with the remaining drugs.

## 3 Discussion

Sweet syndrome is typically classified into three subtypes: classical or idiopathic, malignancy‐associated, and drug‐induced. This classification is not merely descriptive but clinically meaningful, as it guides the search for underlying triggers and determines the optimal management approach. The distinctions among these subtypes have been well established in the literature [1, 3].

In idiopathic Sweet syndrome, episodes are often preceded by infections or autoimmune flares. Malignancy‐associated Sweet syndrome, on the other hand, is most strongly linked to haematological malignancies—particularly acute myeloid leukaemia—where cutaneous flares may parallel disease activity or even herald relapse [3, 6]. Drug‐induced Sweet syndrome typically occurs following exposure to medications such as granulocyte colony‐stimulating factor, antibiotics, or antiepileptics [2, 3]. Antituberculous therapy (ATT), although an extremely uncommon trigger, has been described in isolated case reports [4, 5].

To standardise diagnosis, Su and Liu (1986) and later von den Driesch (1994) proposed diagnostic criteria that remain widely accepted [2]. According to these, a diagnosis of Sweet syndrome requires the presence of both major criteria—namely, the abrupt onset of painful erythematous plaques or nodules, and histopathological evidence of a dense neutrophilic infiltrate without leukocytoclastic vasculitis—together with at least two of four minor criteria: fever above 38°C; an associated malignancy, infection, inflammatory disease, or drug exposure; an excellent response to systemic corticosteroids or potassium iodide; and laboratory abnormalities such as neutrophilia or elevated inflammatory markers.

Our patient clearly fulfilled both major criteria, as demonstrated by typical skin lesions and confirmatory biopsy findings, as well as three minor criteria: fever, a temporal relationship to ATT, and marked neutrophilia accompanied by a dramatic response to corticosteroids. These collectively satisfy the diagnostic definition of Sweet syndrome [2].

The pulmonary involvement in this case further supports the diagnosis of systemic Sweet syndrome, though such manifestations can easily confound clinicians managing tuberculosis patients. New pulmonary infiltrates during ATT are often misattributed to treatment failure, infection, or malignancy. Recognising this inflammatory manifestation is crucial, as timely diagnosis spares patients from unnecessary antimicrobial escalation and leads to rapid clinical improvement with corticosteroid therapy—a pattern consistently observed in previously reported TB‐associated Sweet syndrome cases [4, 5].

In our patient, oral prednisolone was initiated at a dose of 0.5 mg/kg/day (30 mg daily), followed by a taper of 5 mg per week until discontinuation. This regimen aligns with current literature, which recommends starting with 0.5–1.0 mg/kg/day and tapering gradually over 2–6 weeks, typically reducing by 5 mg every 3–5 days, depending on clinical response [1, 7]. Lesions in most cases begin to resolve within 3–10 days, and fever subsides within 48 h of steroid initiation. A slow taper is essential to minimize relapse, which may occur if corticosteroids are withdrawn too rapidly. In recurrent or chronic cases, low‐dose maintenance (e.g., 5–10 mg/day) or steroid‐sparing agents such as colchicine or dapsone may be considered.

The dramatic clinical improvement observed in this patient following corticosteroid initiation strongly supports a drug‐induced inflammatory mechanism rather than an infectious process [6]. Importantly, continued anti‐tuberculosis therapy was possible after confirming rifampicin as the causative agent through structured drug rechallenge, thereby avoiding unnecessary discontinuation of other first‐line drugs.

In conclusion, rifampicin‐induced Sweet syndrome is an exceptionally rare entity but should be suspected in patients who develop painful erythematous plaques while receiving ATT. Exclusion of other causes, particularly haematological malignancies, is crucial. Early recognition and prompt initiation of corticosteroids are highly effective and can prevent unnecessary interruption of tuberculosis treatment.

## Author Contributions

All listed authors contributed to the article.

## Funding

The authors have nothing to report.

## Ethics Statement

The authors declare that written informed consent was obtained for the publication of this manuscript and accompanying images and attest that the form used to obtain consent from the patient complies with the Journal requirements as outlined in the author guidelines.

## Conflicts of Interest

The authors declare no conflicts of interest.

## Data Availability

The data that support the findings of this study are available from the corresponding author upon reasonable request.
